# Genetic Characterization of *Staphylococcus aureus* From Subclinical Mastitis Cases in Dairy Cows in Rwanda

**DOI:** 10.3389/fvets.2021.751229

**Published:** 2021-11-18

**Authors:** Jean Baptiste Ndahetuye, Mikael Leijon, Renée Båge, Karin Artursson, Ylva Persson

**Affiliations:** ^1^Department of Clinical Sciences, Swedish University of Agricultural Sciences, Uppsala, Sweden; ^2^Department of Veterinary Medicine, College of Agriculture, Animal Sciences and Veterinary Medicine, University of Rwanda, Kigali, Rwanda; ^3^National Veterinary Institute, Uppsala, Sweden; ^4^Department of Biomedical Sciences and Veterinary Public Health, Swedish University of Agricultural Sciences, Uppsala, Sweden

**Keywords:** subclinical, core genome multilocus sequence typing, antibiotic resistance, AMR, whole genome sequencing

## Abstract

Whole-genome sequencing was carried out on 30 *Staphylococcus (S.) aureus* isolates from dairy cows with subclinical mastitis from all five provinces of Rwanda. Twenty-five of the isolates produced enough sequence to be analyzed using core genome multilocus sequence typing (cg-MLST). The isolates group into three main clusters. The largest cluster contain isolates of sequence type (ST) 152 (*n* = 6) and the closely related ST1633 (*n* = 2). These sequence types have previously mainly been encountered in humans. The isolates of the second-largest cluster belong to ST5477 (*n* = 5),so far exclusively isolated from cows in Rwanda. The third cluster consists of isolates of ST97 (*n* = 4), which is a well-known bovine-adapted sequence type. These three clusters were all widespread over the country. Isolates of the usually human-adapted sequence types 1 (*n* = 2) and 5 (*n*= 1) were found and a single isolate of ST2430, previously found among humans in Africa. Finally, four isolates of novel sequence types were found: ST7108 (*n* = 2), ST7109 (*n* = 1), and ST7110 (*n* = 1). The blaZ penicillin resistance gene was found in 84% of the isolates and was in all cases corroborated by phenotypic resistance determination. Five (20%) of the isolates carried a tetracycline resistance gene, tet(K) or tetM, and three of these five also displayed phenotypic resistance while two isolates carried a tetM-gene but were yet tetracycline susceptible. Seven (28%) isolates carried the dfrG gene conferring resistance to trimethoprim. Four of these isolates indeed were resistant to trimethoprim while three isolates were sensitive. The str gene conferring resistance to aminoglycosides was found in three isolates; however, none of these displayed resistance to gentamycin. Our data revealed a high diversity of the sequence types of *S. aureus* isolates from cows with subclinical mastitis in Rwanda. Two major clusters of ST97 and ST5477 are likely to be bovine adapted and cause mastitis while the third cluster of ST152 usually have been found in humans and may signify a recent transmission of these types from human to cows, for example from hand milking. The high prevalence of this sequence type among dairy cows may pose zoonotic threat. The sequence types were widely distributed without any geographic correlation. Penicillin resistance, the most common type of resistance with a prevalence over 80%, but also tetracycline and trimethoprim resistance were displayed by several isolates.

## Introduction

Bovine mastitis is a common and costly disease on dairy farms that not only affects milk yields but also milk quality. Typically, mastitis is an inflammation of the mammary gland due to microorganisms invading the udder, but also physical or chemical trauma of the mammary gland could be the origin of udder inflammation.

*Staphylococcus* (*S*.) *aureus* is among the microorganisms that cause both clinical and subclinical mastitis (SCM) and is characterized by its reoccurring and chronic type of mastitis (1). Its contagious nature means that the infected udder becomes a reservoir for the bacterium which is transmitted to uninfected animals in the herd mainly during milking (2). In addition, the pathogen is hard to cure and eradicate in herds because of its ability to persist in cow environment and to colonize skins or mucosal epithelia (3). Reservoirs of *S. aureus* include teat skin, external orifices, housing, feedstuffs, humans, non-bovine animals, air, equipment, bedding, insects, and water (4). The bacteria are spread to uninfected quarters by teat cup liners, milkers' hands, wash cloths, contaminated floor/bedding, and flies.

*S. aureus* is common in mastitis cases in east African countries, where implementation of the ten-point mastitis control plan is still lacking (5–8). However, *S. aureus* is a major cause of mastitis also in developed countries which have successfully implemented mastitis control plans for decades (9, 10). This highlights the need for new ways to study *S. aureus* infection dynamics with the aim to control and limit the infection. Genotyping is one of the ways to understand the characteristics of strains of *S. aureus* for several reasons. For example, the cure rate of mastitis caused by *S. aureus* is very variable and may depend on prevalent genotypes (11). Haveri et al. (12) implied that persistence of mastitis infection depended on genotypes. Furthermore, the virulence and spread of *S. aureus* is also strain dependent (3). Antimicrobial resistance in *S. aureus* is increasingly becoming a problem worldwide, and therefore, it is important to monitor mechanisms of resistance in this pathogen in order to guide therapy and collect knowledge of resistant strains in different ecological niches (13).

DNA-based methods that are used in strain typing such as multilocus sequence typing (MLST) yield standardized results that can be compared across laboratories using databases (14). This molecular typing method has been used to show that some strains more commonly cause mastitis and/or intramammary infections (IMI) than others (15). Whilst MLST has a greater discriminatory power than non-sequencing-based methods, it still only uses seven genes to assess the relatedness between strains of *S. aureus*. Core genome (cg-) MLST is a recently developed method that typically utilizes whole genome sequencing (WGS) and around 2,000-gene target from the core genome, allowing much greater discrimination and reliability when typing and comparing strains. In cg-MLST, table-top sequencing platforms such as Illumina HiSeq can be used to generate WGS data which can then either be assembled *de novo* or mapped to a reference genome for the organism, and the strains can be typed in this way (16). Using cg-MLST, the strains can be grouped into clonal complexes (CCs) based on how many alleles of each of the genes being assessed they share with other strains. This, too, is an assessment of relatedness as the strains within a CC will share a common lineage. In this study, therefore, cg-MLST was used for the bioinformatic analysis of *S. aureus* strains utilizing Ridom SeqSphere+ (Ridom GmbH, Münster, Germany) for determination of the minimum spanning tree describing the relatedness of the strains. To the best of our knowledge, this is the first study carried out to show genetic diversity or relatedness of *S. aureus* from SCM cases in Rwanda.

## Materials and Methods

### *Staphylococcus aureus* Isolates

Thirty *S. aureus* isolates from SCM cases in dairy cows in Rwanda collected from 2016 to 2017 were included in this study. A SCM case was defined as a quarter with score of ≥3 on a 1–5 scale in the California Mastitis Test for milk samples. Six isolates were selected from each of the five provinces (Kigali, Eastern, Northern, Western, and Southern) of Rwanda. From each province, isolates were randomly selected from individual herds as to simulate natural distribution with the exclusion criterium to select only one sample from each farm. No information about antibiotic use was gathered or known. More information about the collection of isolates have been given elsewhere for samples collected in the Kigali province (6) and the other four provinces (7). Once selected, ~1 μL of each of the samples was cultured on 5% bovine blood agar plates and incubated at 37°C overnight. The colonies were evaluated based on the expected *S. aureus* morphology and partial or complete hemolysis. Cultures with non-uniform colonies were sub-cultured and incubated overnight at 37°C to obtain pure cultures. All cultures were stored at 4°C.

### DNA Extraction

Prior to DNA extraction, isolates were cultured on horse blood agar plates to verify their purity. The EZ1 DNA Tissue Kit (Qiagen, Hilden, Germany) was used for DNA extractions. Approximately 1 μL of pure colony from each of the 30 strains was suspended in 180 μL Digestion Buffer G2, plus 20 μL lysozyme (50 mg/mL; Sigma-Aldrich) and 10 μL lysostaphin (5 mg/mL; Sigma-Aldrich) and incubated at 37°C for 1 h and 30 min. Automated DNA extraction was then carried out using the EZ1 Advanced or Advanced XL robot (Qiagen) following the manufacturer's instructions, with a final elution volume of 50 μL. The extracted DNA was immediately stored at −20°C. DNA concentrations were adjusted to the range 5–15 ng/μL suitable for sequencing using a Qubit® 2.0 fluorometric analysis double-stranded DNA high sensitivity kit (Thermo Fisher Science, Massachusetts, United States).

### Sequencing of *S. aureus* Isolates

All library preparation and sequencing was carried out at Clinical Genomics Stockholm facility at Science for Life Laboratory (Stockholm, Sweden) using an Illumina Novaseq 6000 instrument with a S4 flow cell. Twenty-five of 30 samples produced sufficient sequence data for bioinformatic analysis. No further investigations were carried out on the five failed samples. The successfully sequenced samples had a mapping rate in the range 84.5–96.6% to the NCTC 8,325 strain (GenBank accession NC_007795). The percentage of base pairs with a coverage better than 100 were in the range 89.6–93.8.

### Bioinformatics Analysis of Sequences

#### Sequence Assembly and Genotyping

Sequence assembly was carried using the UniCycler pipeline (17). UniCycler employs read error correction and optimizes *de novo* assembly by SPAdes (18). In addition, UniCycler removes errors in the assembly by using pilon (19). The UniCycler, SPAdes, and pilon versions were v0.4.8-beta, 3.13.0, and 1.23, respectively. Minimum spanning trees were calculated by SeqSphere + version 5.1.0 (20) using the assembled contigs obtained from UniCycler for the 25 isolates with the seed genome with GenBank accession NC_002951.2 and the *S. aureus* cg-MLST version 1.3 containing 1,861 loci (https://www.cgmlst.org/ncs/schema/141106/). The criteria for identification were 100% aligned length and 90% identity. For all 25 strains, 1,692 loci were found and used for creating a minimum spanning tree. Strains with <200 different alleles were considered as members of a cluster. Sequence types (STs) are defined by alleles from the following standard set of *S. aureus* MLST genes: *arcC, aroE, gpF, gmk, pta, tpi*, and *yqiL* (21). MLST profiles were determined at the PubMLST.org website (https://pubmlst.org/organisms/staphylococcus-aureus) which is an open-access, curated database that integrate population sequence data with provenance and phenotype information (22).

#### Detection of Antibiotic Resistance Genes

The UniCycler sequences assemblies were used to detect antibiotic resistance genes by utilizing of the Resfinder 3.2 (23) web server (https://cge.cbs.dtu.dk/services/ResFinder/) with an identity and coverage threshold of 90 and 60%, respectively. In addition, the UniCycler assemblies were analyzed with the resistance gene identifier service of the Comprehensive Antibiotic Resistance Database (CARD; https://card.mcmaster.ca/analyze/rgi) to detect antibiotic resistance genes with the search in “Perfect” and “strict” mode only (24). The results using the two databases were consistent except that aminoglycoside resistance only were found with Resfinder. In parallel, the 25 *S. aureus* isolates were tested for antimicrobial susceptibility by determination of minimum inhibitory concentration (MIC) using a micro-dilution method according to recommendations from the Clinical and Laboratory Standards Institute using VetMIC™ panels (SVA, Uppsala, Sweden). Isolates were tested for susceptibility to penicillin, tetracycline, trimethoprim, and gentamicin.

## Results

### Core Genome Multilocus Sequence Typing Results

**Figure 1:**
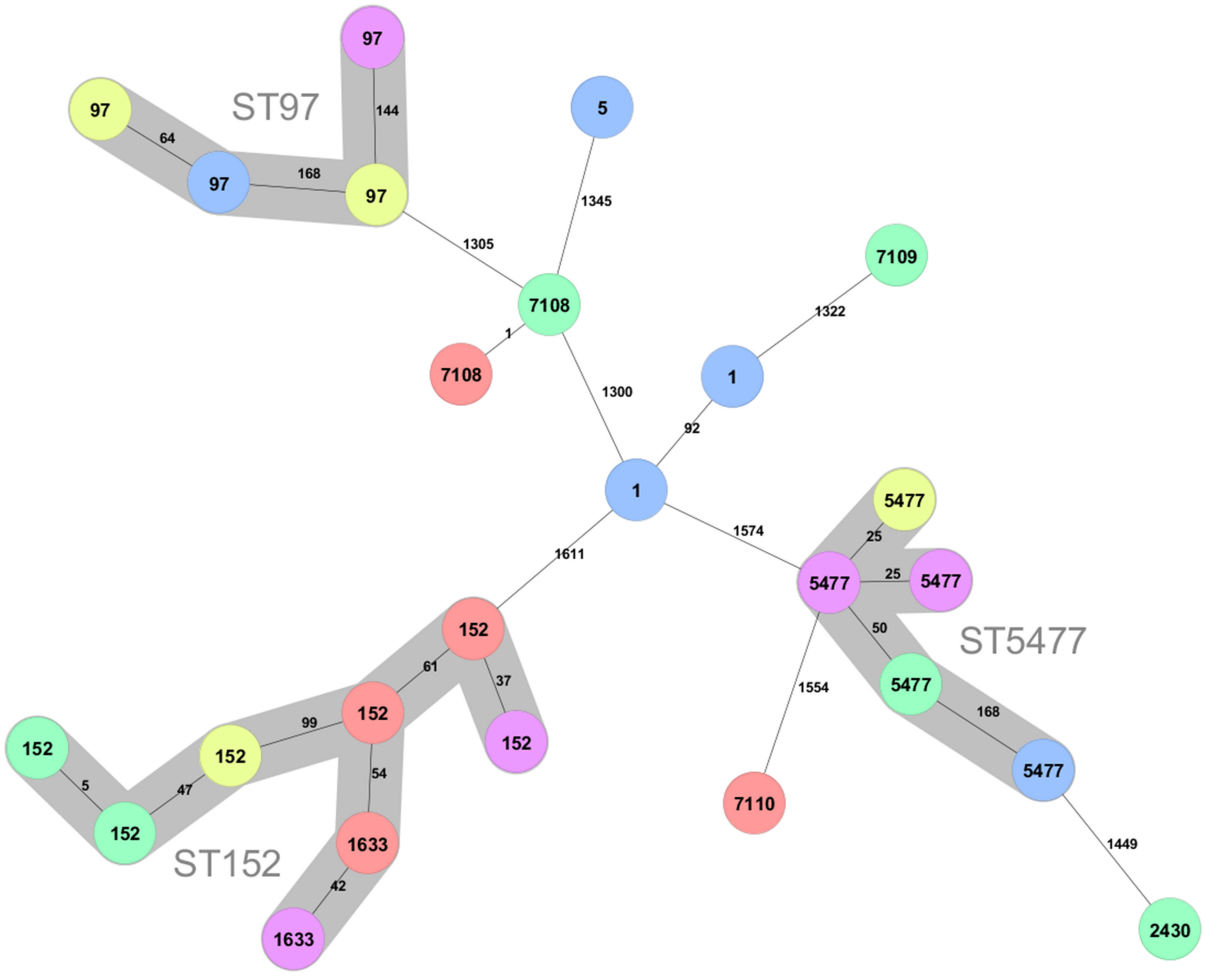
Minimum spanning tree created for the 25 *S. aureus* strains from the Kigali (yellow), Eastern (red), Western (pink), Northern (green), and Southern (blue) province of Rwanda. The tree was created using 1,692 loci. Clusters connecting isolates with less the 200 different loci are indicated with background colors and named with the ST number in the cluster. The ST of the isolates are shown on the nodes and the number of different alleles between pairs of isolates are shown at the connecting lines.

Three main clusters can be discerned among the 25 isolates (Figure 1). The largest cluster contains isolates of sequence type (ST) 152 (*n* = 6) and the closely related ST1633 (*n* = 2), which differ from ST152 in a single allele. The ST152/1663 isolates were found in all provinces except the Southern.

The second largest cluster (*n* = 5) consists of ST5477. The present data indicate that also this sequence type is geographically dispersed in Rwanda since it is found in all provinces except the Eastern.

A third cluster consist of isolates that belong to the well-known bovine adapted CC97 clonal complex (15, 25–28). This sequence type was found in the Kigali, Southern, and Western provinces and thus also display large geographical spread.

There are two isolates of ST1 and one isolate of ST5, which typically are human adapted. In addition, there are four isolates of novel sequence types. Two of these are of ST7108, which differ fromST20 with a single mutation and one isolate each of sequence type ST7109 and ST7110. ST7109 differ from ST101 with a single mutation while ST7110 is most closely related to ST5476, which is a sequence type represented by a single isolate in pubMLST obtained from a mastitis case in Rwanda 2018 (29). All sequence types found in the present study are listed in Table 1.

**Table 1:** Antibiotic resistance profiling of the *S. aureus* strains obtained from subclinical mastitis cases in dairy cows from five regions in Rwanda.

			**Resistance [Genotype (+/–), Phenotype (R/S)]**			
**Strain**	**Province of isolation**	**ST**	**Penicillin** **(blaZ)**	**Tetracycline(tet(K)/tetM)**	**Trimethoprim** **(dfrG)**	**Gentamicin (str)**
JB1	Kigali	97	+, R	+/–, R	–, S	+, S
JB3	Kigali	152	+, R	–/–, R	–, S	–, S
JB4	Kigali	5,477	+, R	+/–, R	–, S	–, S
JB5	Kigali	97	+, R	–/–, S	–, S	–, S
JB7	Eastern	7,108	–, S	–/–, S	–, S	–, S
JB9	Northern	2,430	+, R	–/+, S	+, S	–, S
JB10	Northern	152	+, R	–/–, S	–, S	–, S
JB11	Eastern	152	+, R	–/–, S	+, R	–, S
JB12	Southern	5,477	+, R	–/–, S	–, S	–, S
JB13	Southern	1	–, S	–/–, S	–, S	–, S
JB15	Southern	97	+, R	–/–, S	–, S	–, S
JB16	Southern	1	+, R	–/–, S	–, S	–, S
JB17	Northern	5,477	+, R	–/–, S	–, S	–, S
JB18	Northern	7,109	+, R	–/–, S	–, S	–, S
JB19	Western	1,633	+, R	–/–, S	+, S	–, S
JB21	Southern	5	+, R	–/–, S	+, R	–, S
JB23	Eastern	1,633	+, R	–/–, S	+, R	–, S
JB24	Western	5,477	+, R	–/–, S	–, S	–, S
JB25	Northern	152	+, R	–/–, S	–, S	–, S
JB26	Western	152	+, R	–/–, S	+, R	–, S
JB30	Eastern	7,108	–, S	–/–, S	–, S	–, S
JB31	Western	5,477	+, R	–/–, S	–, S	+, S
JB33	Western	97	+, R	+/–, S	–, S	+, S
JB34	Eastern	152	+, R	–/–, S	+, S	–, S
JB35	Eastern	7,110	–, S	+/–, R	–, S	–, S
	Prevalence (phenotype):		84% (0.7–0.98)	16%(0.02–0.30)	16% (0.02–0.30)	0%

For the phenotypic antibiotic resistance prevalence, a 95% confidence interval is given calculated by:$p\;\pm \;1.96\;*\;\sqrt{p\;*\;\left(p-1\right)/n}$, where p is the sampled prevalence and n is the sample size.

Overall, there is no geographic association that can be discerned for the sequence types of the isolates in the present study.

### Antibiotic Resistance

Results of genotypic and phenotypic resistance determinations are presented in Table 1. The BlaZ gene, which confer penicillin resistance, was prevalent at a level of 84% and was in all cases supported by MIC phenotypic resistance results. Four of the isolates (16%) were resistant to tetracycline. There is not full accordance between the presence of tet(M) or tetK genes and the observed resistance pattern (Table 1). For two tetracycline sensitive isolates, either the tetK or the tet(M) gene is found from the NGS-data, while one resistant isolate lack both genes. The dfrG gene which encodes trimethoprim resistance was present for seven isolates but only four of these actually displayed resistance, thus the prevalence was 16%. The Str genes which encodes resistance among aminoglycosides including gentamicin was found in three isolates, but all isolates were still susceptible to gentamicin.

## Discussion

The present study is of limited scope encompassing only 25 *S. aureus* isolates from SCM cases in Rwanda, but this still has increased the number of *S. aureus* isolates from Rwanda in pubMLST almost tenfold. However, the samples have been collected in roughly equal numbers from all provinces of the country, which allows some important observations to be made even from this limited sample set. It is clear from the cg-MLST data that the isolates mainly fall into three clusters. Not unexpectedly, one cluster belongs to the clonal complex CC97, which is common among cattle all over the world. Four samples, collected in three different provinces, belong to this cluster.

A second, presumably also bovine adapted, cluster is of ST5477. This sequence type has, except for the five isolates of the present study, only been isolated from a mastitis case in the Musanze district in the Northern Province of Rwanda (29). In the same study, *S. aureus* of sequence type ST5476 was isolated from another mastitis case in the same district. This ST has five identical alleles and is most closely related to the novel sequence type ST7110, found in the present work. A second singleton in the present data, also with a ST5477 isolate as the closest neighbor in the minimum spanning tree, is a ST2430, although only distantly related (Figure 1). ST2430 was first discovered when isolated from inpatients in Thika, Kenya in 2014 (30), but was later also found in an isolate from 1995 from a pyomyositis case in Uganda in (31). Single locus variants of this sequence type are found all over the world but invariably from human hosts (31). Thus, the current evidence indicates that there exists a novel bovine clonal complex in the Rwanda region related to ST5477, while ST2430 are more likely a transfer from humans. The ST7110 and ST5476 might represent a second bovine adapted cluster since all entries in pubMLST with three or more alleles identical to ST5476 have been isolated from cows with ST7110 (present study), ST5475 andST5476 isolated in Rwanda, and ST3591 isolated from a milk sample 2009 in Kenya (31).

The third and largest cluster, surprisingly, is constituted of ST152 and ST1633, which is a single locus variant of ST152. ST152 was first isolated from humans in Europe (32) and have subsequently been shown to be an important and prevalent sequence type infecting humans in many African countries (30, 33–41). These ST152 strains with a local predominance in African countries are typically of spa-type t355, Panton-Valentine leucocidin (PVL) positive and methicillin susceptible (MSSA). To our knowledge, infections of bovids with ST152 in Africa have not been reported. However, Mekkonen et al. (42) reported three PVL+ isolates from dairy cows from north-western Ethiopia with spa type t355, which are typical features of ST152 and indeed all eight ST152/ST1633 bovine isolates of the present work carry the lukF-PV and lukS-PV genes signifying PVL positivity, and they are all of spa-type t355 (data not shown). The large prevalence of ST152 (PVL+) among dairy cows in Rwanda is serious since it may pose a public health risk *via* zoonotic transfer of pathogen strains to humans. Since hand milking is still prevalent in Rwanda, it is possible that human ST152 strains initially have infected the cows. These strains were distributed over almost all districts of Rwanda (Table 1), and apparently, bovine ST152 is prevalent all over the country. It can be hypothesized that since management of herds are similar and include hand milking and lack of post milking teat dipping, there will be opportunities for human contact with animals in absence of consistent disinfection. This will facilitate transmission of human adapted pathogens to the dairy cows during milking. Similar management means that pattern of transmission is the same across regions and that is why there were positive identification of ST152/1633 in all regions included in the study. Interestingly, when screening milk and dairy products in southern Italy for MRSA, Basanisi et al. (43) found that PVL encoding ST152 (t355) accounted for 67.5% of all MRSA isolates (*n* = 40). In fact, ST152 isolates have also sporadically been isolated from humans in Europe and are usually methicillin resistant (44–47). It has been suggested that ST152 is an originally African lineage which first acquired PVL and subsequently after introduction to Europe also have acquired methicillin resistance (41). Although the number of isolates is low in the present study, the geographic spread of sampling still makes it very likely that the prevalence of MSSA ST152 (PVL+) in the bovine population of Rwanda is high. Taken together with the locally high prevalence of MRSA ST152 (PVL+) in dairy and milk products in the Apulia region of southern Italy (43), it indicates that ST152 have capacity to establish in the bovine population. Since many investigations only genotype MRSA strains, the possibility exists that MSSA ST152(PVL+) is underdiagnosed both in Europe and Africa. Due to the often-high pathogenicity of PVL+ *S. aureus* strains, it is important to further investigate the epidemiology and prevalence of ST152.

Three novel sequence types were discovered in the present work (Figure 1, Table 1). Besides ST7110, which might represent a bovine adapted complex (see above), the two others are denoted ST7108 and ST7109, which differ with a single mutation from ST20 and ST101, respectively. Two other sequence types represented among the isolates are ST1 and ST5. All these STs have primarily been associated with globally dispersed human infections (26, 27) although they occasionally also are found in bovine isolates (25, 26, 48).

Penicillin resistance was the most common type of resistance with a prevalence over 80%. The high prevalence of penicillin resistance is of concern since it may imply treatment failures of *S. aureus* IMI in dairy cows (49). The prevalence of penicillin resistance found here (84%) for isolates from Rwanda is similar to the 86% that recently was observed for isolates derived from dairy cows in north-western Ethiopia (42). However, tetracycline and trimethoprim resistance were both found among 16% of the isolates (Table 1), which is a significantly lower fraction than the 54 and 79%, respectively, observed in the study from Ethiopia (42). These differences should be interpreted with caution due to the small number of isolates in the present study. The Str genes which encodes aminoglycoside 6-adenylyltransferase and confer resistance among aminoglycosides were found in three isolates, which all, despite this, were susceptible for gentamicin. Since mechanisms of antimicrobial resistance is complex, it is possible to detect resistance genes in susceptible isolates, for example due to lacking, but crucial, accessor genes, or to find phenotypic resistance when there are no resistance genes (13).

In summary, among *S. aureus* isolates collected from milk samples from SCM diagnosed cows, three genotype clusters dominate presumably of bovine-adapted sequence types ST152/1633, ST97, and ST5477. Of these, ST152/1633 may pose a potential zoonotic threat since the sequence type frequently are encountered among humans in Africa while ST5477 so far appear to be a sequence type local to east Africa and Rwanda. Since tetracycline and penicillin are used to treat mastitis in the region (50), one can speculate that their frequent use and lack of biosecurity and mastitis control program in dairy cows in Rwanda (6) have contributed to the high resistance levels reported in this study.

## Data Availability Statement

The datasets presented in this study can be found in online repositories. The names of the repository/repositories and accession number(s) can be found at: NCBI SRA BioProject, accession no: PRJNA767102.

## Ethics Statement

This animal study was reviewed and approved by the Research Screening and Ethics Clearance Committee (RSEC-C) of the College of Agriculture Animal Sciences and Veterinary Medicine, University of Rwanda (UR-CAVM).

## Author Contributions

JN, YP, RB, and KA planned the study. JN carried out the sampling and sample preparations. ML performed bioinformatic analysis of sequence data. JN and ML wrote the manuscript that was revised by KA, RB, and YP. All authors read and approved the final manuscript.

## Funding

The authors would like to acknowledge funding from the Swedish International Development Agency (SIDA), within the University of Rwanda-Sweden programme for research, higher education and institutional advancement, subprogram agricultural sciences, project no. 20290000. Authors would also like to acknowledge the generous support from the United States Agency for International Development (USAID) and its Feed the Future Innovation Lab for Livestock Systems managed by the University of Florida and the International Livestock Research Institute. The contents are the responsibility of the authors and do not necessarily reflect the views of USAID or the United States Government.

## Conflict of Interest

The authors declare that the research was conducted in the absence of any commercial or financial relationships that could be construed as a potential conflict of interest.

## Publisher's Note

All claims expressed in this article are solely those of the authors and do not necessarily represent those of their affiliated organizations, or those of the publisher, the editors and the reviewers. Any product that may be evaluated in this article, or claim that may be made by its manufacturer, is not guaranteed or endorsed by the publisher.
